# Altered Diastolic Flow Patterns and Kinetic Energy in Subtle Left Ventricular Remodeling and Dysfunction Detected by 4D Flow MRI

**DOI:** 10.1371/journal.pone.0161391

**Published:** 2016-08-17

**Authors:** Emil Svalbring, Alexandru Fredriksson, Jonatan Eriksson, Petter Dyverfeldt, Tino Ebbers, Ann F. Bolger, Jan Engvall, Carl-Johan Carlhäll

**Affiliations:** 1 Department of Medical and Health Sciences, Division of Cardiovascular Medicine, Linköping University, Linköping, Sweden; 2 Center for Medical Image Science and Visualization (CMIV), Linköping University, Linköping, Sweden; 3 Department of Medicine, University of California San Francisco, San Francisco, California, United States of America; 4 Department of Clinical Physiology, Department of Medical and Health Sciences, Linköping University, Linköping, Sweden; University of Washington, UNITED STATES

## Abstract

**Aims:**

4D flow magnetic resonance imaging (MRI) allows quantitative assessment of left ventricular (LV) function according to characteristics of the dynamic flow in the chamber. Marked abnormalities in flow components’ volume and kinetic energy (KE) have previously been demonstrated in moderately dilated and depressed LV’s compared to healthy subjects. We hypothesized that these 4D flow-based measures would detect even subtle LV dysfunction and remodeling.

**Methods and Results:**

We acquired 4D flow and morphological MRI data from 26 patients with chronic ischemic heart disease with New York Heart Association (NYHA) class I and II and with no to mild LV systolic dysfunction and remodeling, and from 10 healthy controls. A previously validated method was used to separate the LV end-diastolic volume (LVEDV) into functional components: direct flow, which passes directly to ejection, and non-ejecting flow, which remains in the LV for at least 1 cycle. The direct flow and non-ejecting flow proportions of end-diastolic volume and KE were assessed. The proportions of direct flow volume and KE fell with increasing LVEDV-index (LVEDVI) and LVESV-index (LVESVI) (direct flow volume r = -0.64 and r = -0.74, both P<0.001; direct flow KE r = -0.48, P = 0.013, and r = -0.56, P = 0.003). The proportions of non-ejecting flow volume and KE rose with increasing LVEDVI and LVESVI (non-ejecting flow volume: r = 0.67 and r = 0.76, both P<0.001; non-ejecting flow KE: r = 0.53, P = 0.005 and r = 0.52, P = 0.006). The proportion of direct flow volume correlated moderately to LVEF (r = 0.68, P < 0.001) and was higher in a sub-group of patients with LVEDVI >74 ml/m^2^ compared to patients with LVEDVI <74 ml/m^2^ and controls (both P<0.05).

**Conclusion:**

Direct flow volume and KE proportions diminish with increased LV volumes, while non-ejecting flow proportions increase. A decrease in direct flow volume and KE at end-diastole proposes that alterations in these novel 4D flow-specific markers may detect LV dysfunction even in subtle or subclinical LV remodeling.

## Introduction

Flow in the large vessels and cardiac chambers results from the interplay of multiple important aspects of normal and pathophysiological function, including chamber configuration, material properties, load and contractility. Quantification of 4D cardiovascular flow with phase-contrast magnetic resonance (4D flow MRI) is a versatile approach to the investigation of many cardiovascular disorders where disruption of normal flow can be anticipated due to changes in one or more of those aspects [1]. Many 4D flow MRI applications revolve around vascular territories including the cerebral and pulmonary vasculature and aorta [2–5]. The heart’s more complicated chamber configuration and dynamics of contraction and relaxation present particular challenges that have required the development of novel quantitative parameters for measuring the intracardiac flow. To date, proportions of the functional flow components, kinetic energy and flow structures including vortices inside the chambers have been utilized to investigate left and right ventricular and atrial flow [6–14]. In this way, 4D flow MRI is expanding the assessment of ventricular function beyond conventional parameters including ejection fraction and diastolic filling patterns.

Early work with 4D flow MRI suggested that marked abnormalities in 4D flow volume and energy occurred in severely dilated and functionally depressed left ventricles (LV), wherein the portion of flow that passes directly through the LV in a single cardiac cycle diminishes, with a concomitant increase in the volume and end diastolic kinetic energy (KE) of the non-ejecting volume [15]. Similar findings were subsequently confirmed in a cohort of well compensated dilated cardiomyopathy patients with moderate remodeling and depression of systolic function compared to a cohort of healthy subjects [6].

The determinants and impact of the changes in flow are uncertain, but it has been suggested that increased volume and deceleration of non-ejecting flow may be both an indicator as well as a driver of progressive remodeling [6, 16]. We hypothesized that these potentially adverse changes in flow volume and kinetic energy measured with 4D flow MRI would be detectable early in the course of disease, even in patients with only subtle LV dysfunction and enlargement.

## Methods

### Study population

The patients were enrolled from outpatients at the Department of Cardiology, Linköping University Hospital, as a local sub-study of the EU FP 7 research project DOPPLER-CIP [17]. A total of 26 patients (female, n = 10; male, n = 16) diagnosed with chronic ischemic heart disease were included in the study (Table 1). Presenting symptoms were stable angina, fatigue and dyspnea corresponding to NYHA I and II. 24 patients had objective signs of myocardial ischemia in terms of pathological stress test results (myocardial perfusion scintigraphy, ECG exercise testing or stress echocardiography). The remaining 2 patients were followed at the post infarct clinic due to earlier myocardial infarction, but had no current objective signs of myocardial ischemia in terms of pathological stress test results. In addition, 10 healthy controls with no history of prior or current cardiovascular disease or cardiac medication and a normal echocardiogram were included (Table 1). Exclusion criteria for all patients were: 1) concomitant significant disease or non-cardiological therapy anticipated to impact cardiac remodeling or function, or to make two-year survival unlikely; 2) NYHA III or IV; 3) acute coronary syndrome (STEMI or non-STEMI) in the preceding three months; 4) more than mild valvular disease; 5) atrial fibrillation; 6) significant pulmonary disease; 7) submaximal performance on cardiopulmonary exercise testing; 8) contraindications for CMR. After data analysis, data sets were excluded as follows: 9) inadequate 4D flow MRI flow data quality defined as >10% difference between LV inflow and outflow. In total 14 patients were excluded.

**Table 1:** Characteristics of the study population.

	Control group	Patients	P-value
	n = 10	n = 26	
Age (y)	62 ± 11	68 ± 5	0.068
Gender (f/m)	7 / 3	10 / 16	-
Height (cm)	170 ± 8	170 ± 8	0.771
Weight (kg)	68 ± 9	77 ± 12	0.038
Body mass index	24 ± 2	26 ± 3	0.017
HR at rest (bpm)	72 ± 8	67 ± 10	0.321
Systolic blood pressure (mmHg)	128 ± 12	144 ± 11	0.001
Diastolic blood pressure (mmHg)	76 ± 6	78 ± 7	0.417
VO_2max_/bw (ml/(min·kg))	-	22 ± 6	-
**MRI data**			
Ejection fraction (%)	64 ± 6	61 ± 7	0.213
LVEDV (ml)	133 ± 21	126 ± 30	0.517
LVEDVI (ml/m^2^)	74 ± 9	66 ± 13	0.079
LVESV (ml)	48 ± 10	45 ± 15	0.548
LVESVI (ml/m^2^)	27 ± 5	23 ± 7	0.194
LVMI (g/m^2^)	-	52 ± 11	-
Positive LGE (number of patients)	-	10	-
**NYHA classification (number of patients)**			
I	-	16	-
II	-	10	-
**Medication (number of patients)**			
Beta-blocker	0	16	-
ACE-I	0	7	-
ARB	0	6	-
Calcium channel blocker	0	9	-
Nitrate	0	9	-
Aspirin	0	22	-
Clopidogrel	0	2	-
Warfarin	0	1	-
Statin	0	23	-
Diuretic	0	2	-

ACE-I, angiotensin converting enzyme inhibitor; ARB, angiotensin receptor blocker; HR, heart rate; LVEDV, left ventricular end diastolic volume; LVEDVI, left ventricular end diastolic volume index; LVESV, left ventricular end systolic volume; LVESVI, left ventricular end systolic volume index; LVMI, left ventricular mass index; NYHA, New York Heart Association; Positive LGE, late gadolinium enhancement in the LV myocardium; VO_2max_/bw, maximal oxygen consumption/body weight.

The research complied with the declaration of Helsinki. Written consent was obtained from all participants and the study, including the consent procedure, was approved by the Regional Ethical Review Board in Linköping.

### Data acquisition

#### Magnetic resonance imaging

MRI examination was performed to acquire 4D velocity data, morphological images, and late gadolinium enhancement (LGE) images on a clinical 3T Philips Ingenia scanner (Philips Healthcare, Best, the Netherlands) at the Center for Medical Image Science and Visualization (CMIV), at the University of Linköping, Sweden. The three-directional, three-dimensional, cine phase-contrast CMR velocity data were acquired during free- breathing gradient-echo pulse-sequence with interleaved three-directional flow-encoding and retrospective vector cardiogram controlled cardiac gating. Data were acquired at end-expiratory phase using navigator gating. Scan parameters included: velocity encoding (VENC) = 120 cm/s, flip angle = 10°, echo time = 2.6 ms, repetition time = 4.3 ms, parallel imaging (SENSE) speed up factor = 3, k-space segmentation factor = 3, spatial resolution 2.8 x 2.8 x 2.8 mm, k-space acquisition: Cartesian with elliptic centric filling ("cut corner"), acquired temporal resolution 52.8 ms. The field of view was adjusted to cover the whole heart for each subject. Scan time was 7–9 min excluding and approximately 15 min including the navigator efficiency at heart rate 60 bpm. Concomitant gradient field effects were corrected on the scanner [18]. Cine balanced steady-state free-precession (bSSFP) was used to acquire 2-, 3-, and 4-chamber long axis and a stack of short-axis images at 30 time frames during end expiratory breath holds. The short- and long-axis images had an acquired resolution of 1.0 X 1.0 mm and a slice thickness of 8.0 mm. LGE images were acquired approximately 20 min after the administration of gadopentetate dimeglumine (Gd-DTPA) 0.2 mmol/kg bodyweight (Bayer Healthcare, Berlin, Germany).

#### VO_2max_ testing

Maximal cardiopulmonary exercise tests were performed with an electrically braked bicycle ergometer in purpose of describing the study population. Data for gas analysis were collected using Jaeger Oxycon-Pro (Viasys Healthcare, Hoechberg, Germany). The tests were terminated on indications of maximal effort: oxygen consumption leveling off and respiratory exchange ratio (RER) > 1.0. The VO_2max_ value was estimated by taking the mean of the two largest values and was set in relation to body weight (bw).

### Data analysis

The 4D flow data were post-processed and analyzed using in-house software as previously published [18]. The LV chamber was manually segmented at times of end-diastole (ED) and end-systole (ES) using the freely available software Segment (version 1.9. Medviso, Lund, Sweden) [19]. ED was defined as the time frame after mitral valve closure and ES as the time frame after aortic valve closure. The segmentation was performed to cover the complete left ventricular intra-cavitary blood volume and thereby include as many flow voxels as possible. The most basal plane at ES was defined as the plane closest to the atrioventricular plane that did not include any left atrial volume.

**Fig 1:**
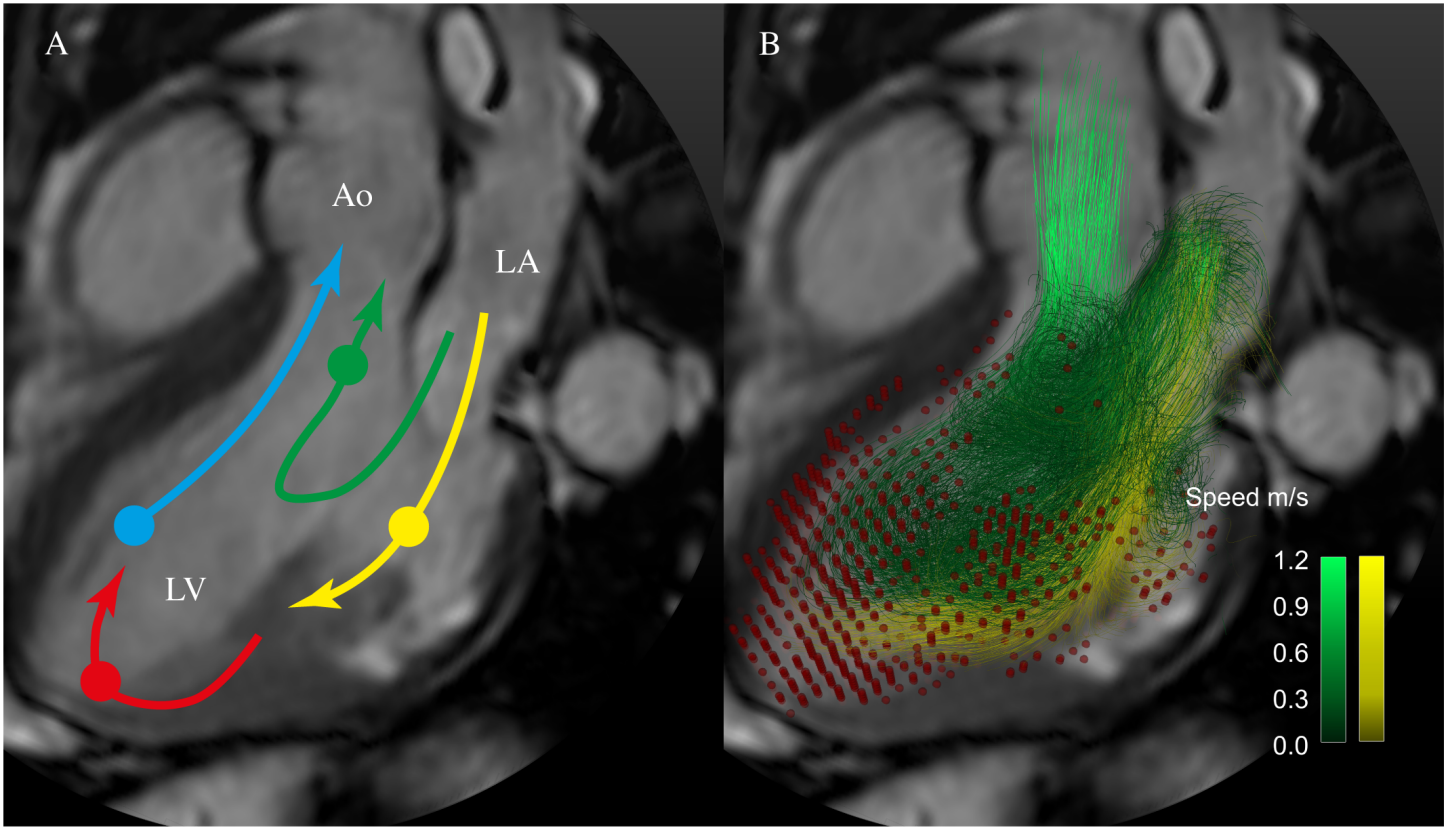
4D flow components. A. Schematic of the routes of the four LV flow components; direct flow (green), retained inflow (yellow), delayed ejection flow (blue), and residual volume (red). A semitransparent grayscale three-chamber image provides morphological orientation. Circles indicate the approximate location of the center of mass of each component at the time of end-diastole. B. Particle trace pathlines indicate routes of direct flow (green) and retained inflow (yellow). Red dots indicate the positions of the residual volume pathlines at end-diastole. Non-ejected flow comprises the retained inflow and the residual volume. Ao, aorta; LA, left atrium; LV, left ventricle.

The end diastolic LV blood volume was separated into four functional flow components, based on the blood´s transit through the LV chamber, using a previously evaluated method [18, 20]: direct flow (DF) = blood that both enters and leaves the LV during one cardiac cycle, retained inflow = the volume that enters the LV but does not leave during the same cardiac cycle, delayed ejection flow = the volume that was in the LV in the beginning of the cycle and which is ejected during the cycle, and the residual volume = the volume that neither enters nor leaves the LV during the cardiac cycle (Fig 1A). The non-ejecting (NE) volume of the heart was the combined retained inflow and residual volume (Fig 1B). The volume of the flow components was expressed as a proportion of the LVEDV (DF/LVEDV volume-ratio, and NE/LVEDV volume-ratio, respectively). Data sets were subjected to quality check. Data sets with >10% discrepancy between LV inflow and outflow were excluded and there was no significant difference in LV inflow volume versus outflow volume (67 ± 12 vs 66 ± 13 ml, P = 0.871) in the included data sets. LV inflow and outflow were taken from the pathline analysis. LV inflow = Direct flow + Retained inflow and outflow = Direct flow + Delayed ejection flow. In this way the quality of the whole LV blood pool is assessed. Flow data was also visually inspected in EnSight Standard version 10.0.3(b) (EnSight, CEI Inc, Research Triangle Park, NC, USA) for signs of aberrant traces that would indicate poor data quality; representations of individual ventricular flow were constructed in EnSight.

The kinetic energy (KE) for the volume of each flow component and for the entire LVEDV, respectively, was calculated over the cardiac cycle by using the density of blood (ρ_blood_ = 1060 kg/m^3^) and a known velocity at every point in time for all pathlines in the volume. $KE\;=\;\frac{1}{2}\times \;\rho_{blood}\;\times \;V_{pathline}\;\times \;v_{pathline}$^2^, where V_pathline_ is the volume that one pathline represents and v_pathline_ is the velocity of the pathline at a given point in time [6]. The KE of each component’s blood volume at end-diastole was expressed as a total amount. The proportion of each component’s KE relative to the KE of the total end-diastolic volume was calculated (DF/LVEDV KE—ratio and NE/LVEDV KE-ratio).

LVEDV, LVEF, LVESV and LV mass were assessed from clinically segmented morphological CMR images. LVEDV, LVESV and LV mass were indexed to Body Surface Area (BSA) according to the Mosteller formula.

In sub-analyses, the patient cohort was stratified into two subgroups according to either LVEDV index (LVEDVI with cutoff value of 74 ml/m^2^ [21, 22] or presence or absence of positive LGE test.

### Statistical analysis

LV volume parameters were correlated with LV 4D flow measures using Pearson´s parametric correlation analysis. Clinical parameters were compared using t-test. In cases where data had a non-Gaussian distribution Mann—Whitney U test was used. The difference between LV inflow and outflow volumes was analyzed using t-tests for paired observations. One way Anova analysis with Tukey´s post hoc test was used to compare the patient subgroups based on LVEDVI or LGE and the healthy controls. Data are presented as mean ± SD and a P-value <0.05 was considered statistically significant. The software STATISTICA (StatSoft, Inc. 2011 version 10) was used to perform the statistical analyses.

## Results

### Clinical parameters

The patient group had higher BMI and systolic blood pressure compared to the healthy control group (Table 1). The patients had a mean age of 68 ± 5 years and presented a range of normal to mild LV remodeling (mild LV remodeling was defined by LVEDVI >90 ml/m^2^ and LVESVI >30 ml/m^2^), normal to mildly depressed LV systolic function (mild LV systolic dysfunction was defined by LVEF <59%), and normal to moderately depressed maximal oxygen consumption (VO_2max_/bw, 22 ± 6 ml / (min · kg)). Ten subjects had evidence of LV myocardial damage based on positive late gadolinium enhancement; none of the subjects had more than NYHA Class II symptoms.

### Correlation analyses

**Fig 2:**
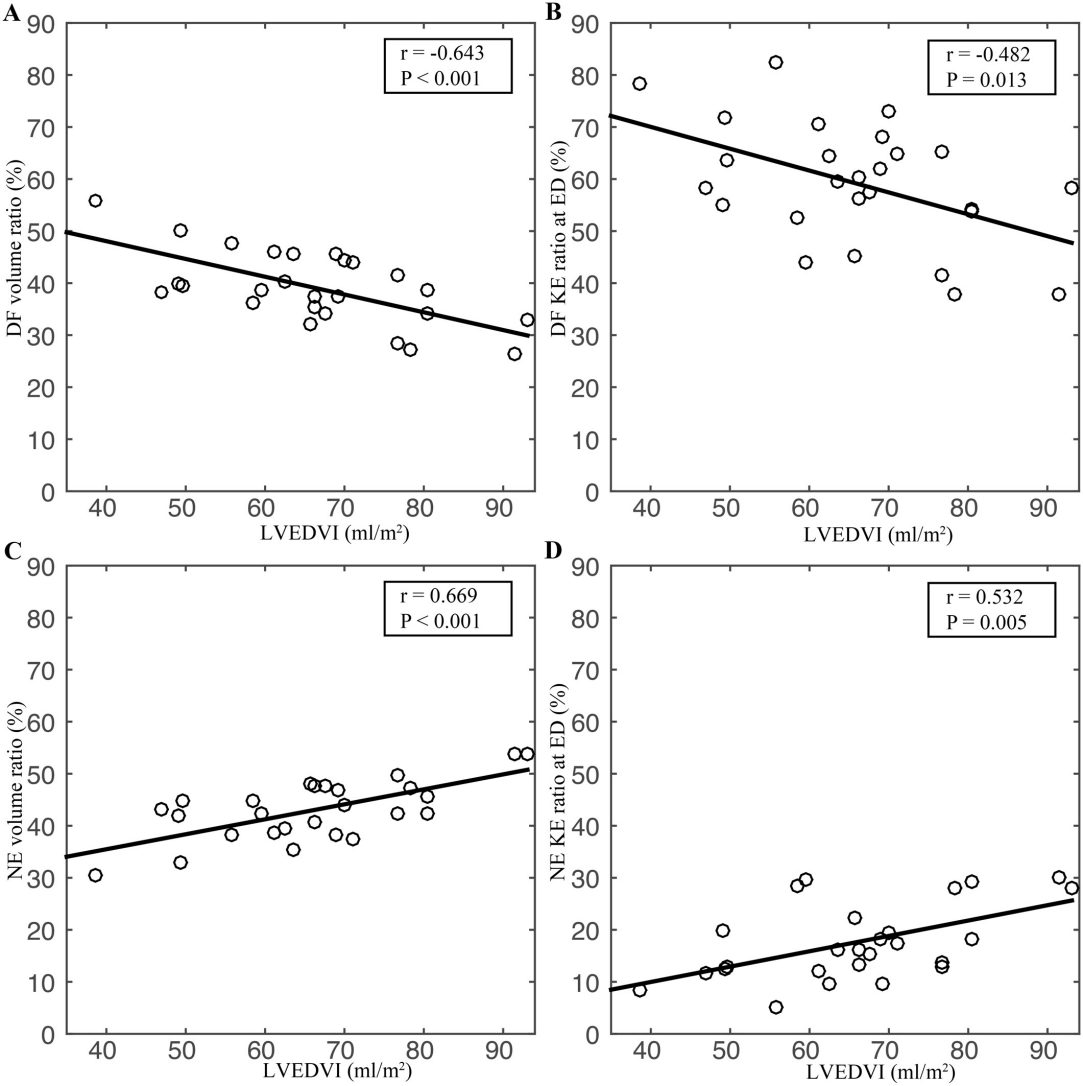
Correlation plots. A. Direct flow (DF) volume ratio (in relation to LVEDV) versus LVEDV index (LVEDVI). B. DF Kinetic energy (KE) ratio at ED versus LVEDVI. C. Non-ejecting (NE) volume ratio versus LVEDVI. D. NE KE ratio at ED versus LVEDVI.

With increasing LVEDV, the volumes of all four LV flow components increased (r = 0.64, 0.77, 0.75 and 0.86 for direct flow, delayed ejection, retained inflow and residual volume, respectively; P<0.005 for all four comparisons). The proportion of the direct flow volume relative to the total LVEDV (DF volume ratio) fell with increasing LVEDVI and LVESVI (Fig 2A, Table 2). The proportion of direct flow KE relative to the total LVEDV KE at end diastole (DF KE ratio) fell with increasing LVEDVI and LVESVI (Fig 2B, Table 2).

**Table 2:** Correlation between 4D flow measures and LV volumes.

4D flow	LV volumes	r-value	P-value
DF Volume ratio	LVEDVI	-0.64	<0.001
DF Volume ratio	LVESVI	-0.74	<0.001
DF KE ratio	LVEDVI	-0.48	0.013
DF KE ratio	LVESVI	-0.56	0.003
NE Volume ratio	LVEDVI	0.67	<0.001
NE Volume ratio	LVESVI	0.76	<0.001
NE KE ratio	LVEDVI	0.53	0.005
NE KE ratio	LVESVI	0.52	0.006

DF, direct flow; KE, kinetic energy at end diastole; NE, non-ejecting; LVEDVI, left ventricular end-diastolic volume index; LVESVI, left ventricular end-systolic volume index.

The proportion of the volume of the non-ejecting components relative to the total LVEDV (NE volume ratio) increased with LVEDVI and LVESVI (Fig 2C, Table 2). The proportion of KE of the non-ejecting components at ED relative to the total LVEDV KE (NE KE ratio) increased with LVEDVI and LVESVI (Fig 2D, Table 2).

The DF Volume ratio and DF KE ratio correlated positively to LV EF (r = 0.68, P < 0.001 and r = 0.47, P < 0.05, respectively). NE volume ratio and NE KE ratio correlated negatively to LV EF (r = -0.74, P < 0.001 and r = -0.44, P < 0.05, respectively).

### Sub group analyses

**Fig 3:**
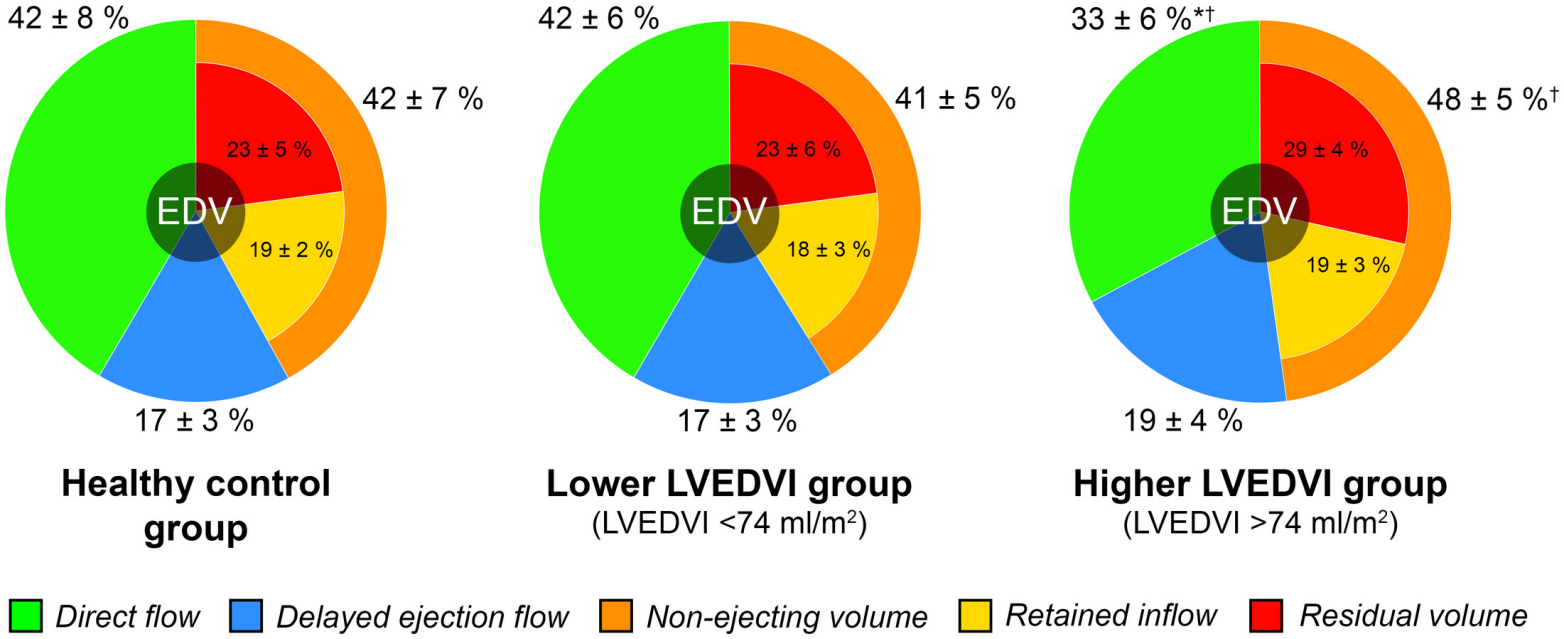
The 4D flow components as proportions of total LV EDV (percent ± SD) in the three sub-groups stratified by LVEDVI. Non-ejecting volume is comprised of the Retained inflow (yellow) and Residual volume (red) flow components (yellow + red = orange). * P<0.05 vs Control group; † P<0.05 vs Lower LVEDVI group.

Patients in the higher LVEDVI group had significantly lower DF Volume ratio (0.33 ± 0.06) compared to the patients in the lower LVEDVI group (0.41 ± 0.06) and the healthy controls (0.42 ± 0.08), P < 0.05 for both (Fig 3, Table 3). The patients in the higher LVEDVI group also had lower DF KE ratio (0.50 ± 0.11) and higher NE Volume ratio (0.48 ± 0.05) compared to the lower LVEDVI group (0.63 ± 0.10) and (0.41 ± 0.05) respectively, P < 0.05 for both. Patients in the higher LVEDVI group had lower LV EF (56 ± 4%) compared to the healthy controls (64 ± 6%), P < 0.05.

**Table 3:** Sub-analysis with patients stratified according to LVEDV index.

	Control group	Lower LVEDVI group	Higher LVEDVI group
	n = 10	n = 19	n = 7
**DF Volume ratio**	0.42 ± 0.08	0.42 ± 0.06	0.33 ± 0.06*†
**DF KE ratio**	0.60 ± 0.14	0.63 ± 0.10	0.50 ± 0.11†
**NE Volume ratio**	0.42 ± 0.07	0.41 ± 0.05	0.48 ± 0.05†
**NE KE ratio**	0.19 ± 0.13	0.16 ± 0.06	0.23 ± 0.08
**LV EF (%)**	64 ± 6	62 ± 7	56 ± 4*

DF, direct flow; KE, kinetic energy at end diastole; Higher LVEDVI group, >74 ml/m^2^; Lower LVEDVI group, <74 ml/m^2^; LVEDVI, left ventricular end-diastolic volume index; LV EF, left ventricular ejection fraction; NE, non-ejecting.

* P<0.05 vs Control group;

^†^ P<0.05 vs Lower LVEDVI group.

No intergroup differences were seen between the groups stratified according to LGE and the healthy control group (Table 4).

**Table 4:** Sub-analysis with patients stratified according to Late Gadolinium Enhancement test.

	Control group	Negative LGE	Positive LGE
	n = 10	n = 16	n = 10
**DF Volume ratio**	0.42 ± 0.08	0.41 ± 0.07	0.37 ± 0.07
**DF KE ratio**	0.60 ± 0.14	0.62 ± 0.11	0.55 ± 0.12
**NE Volume ratio**	0.42 ± 0.07	0.42 ± 0.06	0.45 ± 0.05
**NE KE ratio**	0.19 ± 0.13	0.16 ± 0.07	0.20 ± 0.07
**LV EF (%)**	64 ± 6	62 ± 6	59 ± 7

DF, direct flow; KE, kinetic energy at end diastole; LGE, late gadolinium enhancement in the LV myocardium; LV EF, left ventricular ejection fraction; NE, non-ejecting.

## Discussion

This study of quantitative measures of LV flow component volumes and end diastolic kinetic energy in patients presenting a range of normal to mild LV remodeling and normal to mildly depressed LV systolic function, fills a gap in the spectrum of heart failure patients studied with 4D flow MRI to date [6, 15]. A clearer picture emerges when these results are combined with prior work [6]: the proportion of the most efficient component of the LV volume, the direct flow, diminishes with increased LV volumes. At the same time, the amount and proportion of the non-ejecting components of LVEDV increase.

The method for separation of end-diastolic LV volume into functional components with 4D flow MRI was originally developed in response to the hypothesis that, in normal hearts, there would be a consistent distribution of component volumes, their routes and energetics [15]. Among the four functional components of the LV diastolic volume, the direct flow has the most direct route and fastest transit through the LV and best preserves the kinetic energy from inflow to pre-systole. By pre-systole, the predominant trajectory of the direct flow is towards the left ventricular outflow tract, therefore the preserved KE of this flow component may imply less work required for ejection of the stroke volume. The retained inflow and the residual volume in combination constitute the non-ejecting portion of LV volume. As this volume does not leave the ventricle during the ensuing systole, its KE does not contribute directly to ejection, but its distribution and velocity may facilitate ejection by creating an efficient route for the stroke volume through the diastolic LV. This work and prior studies in patients [6, 15, 16] demonstrate that in remodeled, dysfunctional ventricles, there is a shift whereby the volume and KE of the direct flow diminish, while that of the non-ejecting components increase. This shift may potentially serve both as a marker and a driver of disease progression.

LV remodeling and dysfunction are progressive. Detection at its earliest, asymptomatic stages is a clinical priority in order to reduce patient morbidity and mortality, especially since LV dysfunction can be present long before clinical manifestations [23]. The results of this study support our hypothesis that abnormal 4D flow parameters would correlate with subtle LV enlargement, and would be detected even in patients with only subtle or subclinical LV impairment. As in more severely affected ventricles, the 4D flow-based measures studied here confirm the shift from direct to non-ejecting volume and KE, albeit to a lesser degree. Whether progressive changes in the amounts and proportions of ejecting and NE volumes may be able to predict progression or define clinically relevant thresholds along the transition to more severe remodeling and eventual failure will require further study.

In the sub group analyses the direct flow volume was lower in the higher LVEDVI group compared to both the lower LVEDVI group and the controls. On the other hand, also LVEF was lower in the higher LVEDVI group compared to the controls whereas no difference was observed between the two patient sub groups. Although the direct flow volume and KE correlated at least moderately to LVEF, which adds to the validity of the 4D flow method, these parameters are not interchangeable. The LV ejected volume constitutes both direct flow and delayed ejection flow. Direct flow parameters may reflect more aspects of diastolic-systolic coupling than delayed ejection flow and thus LVEF, particularly at these subtle stages of LV dysfunction. Further, 4D flow based KE measures may better track efficiency or potential for excess diastolic pressure than measures merely based on volume; the shift in KE from direct flow to non-ejecting volume components may contribute to higher intraventricular pressure due to deceleration of flow. Eventual comparison of 4D flow MRI parameters to other metrics of ventricular work and demand would be required to confirm their accuracy in reflecting ventricular efficiency.

Approximately 4 out of 10 of the patients had positive LGE and the patterns had a subendocardial origin and were consistent with ischemic heart disease. In the sub-group analyses, however, no differences were observed in flow-specific measures or LVEF between the LGE positive group compared to the LGE negative patients group and the controls. Both the 4D flow specific measures and LVEF reflect more global than regional aspects of LV configuration and function, this in combination with the fact that LGE positive areas had relative small extension/transmurality could explain these findings. Of note, we plan to make further developments to our analysis method and create new flow parameters that more take into account regional aspect of flow.

## Limitations

The present study was based on a relatively small cohort. However, in the research field of intracardiac 4D flow, the number of patients would be considered fairly large. Nevertheless, the study must be viewed as a pilot study bridging earlier research within the field. The lack of longitudinal data constitutes a limitation when assessing the prognostic significance of the 4D flow specific parameters for clinical outcome, and future follow-up studies are needed in order to evaluate this. The present 4D flow findings relate only to subjects in supine position at rest and in sinus rhythm, without significant valvular dysfunction. It is reasonable to assume that different flow patterns would be seen with varying heart rates and different loading conditions, as well as with altered rhythm or valve disease. The morphologic and 4D velocity data were acquired in two consecutive acquisitions; thus, there is a risk of mismatch due to patient movement. This issue was addressed by acquiring morphological data both before and after the 4D velocity data. Although a higher temporal resolution than 52.8 ms would have been preferable, we believe that the current resolution allows acquisition of velocities corresponding to the dominant diastolic flow events.

## Conclusion

Across a spectrum from normal to mildly remodeled ventricles, 4D flow measures of volume and end diastolic kinetic energy demonstrated a progressive shift in the proportions of the direct flow and non-ejecting flow components. Decreased volume and kinetic energy of the direct flow component in this population, which falls between healthy subjects and the cohorts of prior studies of patients with more marked remodeling, suggest that alterations in these novel flow-specific markers may detect LV dysfunction even in subtle or subclinical LV enlargement. The ability to evaluate shifts in the proportions of flow components and their respective kinetic energy characteristics may provide distinct pathophysiological insights relevant to both diagnosis and therapy of heart failure, and empower the detection of ventricular dysfunction in clinically compensated patients.

## Supporting Information

S1 File Underlying data file. (XLSX)
